# Comparison between residents with a 6-year medical program and a 7-year medical program in terms of objective structured clinical examination performance in postgraduate year training in Taiwan: a 2-group pre- and post-test non-synchronized study

**DOI:** 10.3352/jeehp.2022.19.13

**Published:** 2022-06-24

**Authors:** Ya-Ting Chang, Ying-Ying Yang, Chung-Pin Li, Chen-Huan Chen

**Affiliations:** 1 Department of Medical Education, Taipei Veterans General Hospital, Taipei, Taiwan; 2 Department of Family Medicine, Taipei Veterans General Hospital, Taipei, Taiwan; 3 College of Medicine, National Yang Ming Chiao Tung University, Taipei, Taiwan; 4 Clinical Innovation Center, Taipei Veterans General Hospital, Taipei, Taiwan; Hallym University, Korea

**Keywords:** Clinical competence, Curriculum, Clinical reasoning, Medical schools, Taiwan

## Abstract

**Purpose:**

In 2013, medical schools in Taiwan implemented a 6-year medical program that replaced the previous 7-year medical education program. The postgraduate year (PGY) program was also extended from 1 year to 2 years. The new program is characterized by diversified teaching, integration of medical skills, a system-oriented curriculum, and the implementation of primary care and clinical thinking training. The purpose of this study was to examine whether postgraduate residents who learned under the new program have better patient care skills than those who learned under the previous program.

**Methods:**

Of 101 residents in the PGY program at Taipei Veterans General Hospital, 78 were trained in the 6-year program, while 23 were trained in the 7-year program. During the PGY training, 2 objective structured clinical examinations (OSCEs) were used to evaluate clinical reasoning, communication skills, and procedural skills at the beginning of the training and after 11 months of training, respectively. The scores of each OSCE and the rate of improvement of the pre- and post-tests were analyzed.

**Results:**

Residents trained in the new program scored higher on clinical reasoning (P<0.001) and the total scores of the 3 tested skills (P=0.019) on the pre-test. In terms of improvement, residents educated in the previous system improved more in clinical reasoning than those educated in the new education system.

**Conclusion:**

The new medical education program, which emphasizes clinical thinking, improved residents’ clinical skills. The PGY program was effective in improving the clinical performance of residents who were educated in the previous system.

## Introduction

### Background/rationale

In 2013, Taiwan revolutionized its medical education system by changing the medical school curriculum; it replaced the previous 7-year medical education program with a 6-year program [1]. The introduction eliminated the problem of unclear rights and responsibilities associated with the internship in the seventh year. The new system implements the essence of primary patient care taught in the seventh year of the curriculum into the fifth and sixth years of the new system. Unlike the previous system, which divided the medical curriculum into basic science and clinical medicine, the new system placed more emphasis on an integrated curriculum and clinical skills training [1]. Similar academic structure reforms have been observed in other countries [2,3]. However, there is a paucity of literature assessing the differences in performance between students receiving previous and new programs. Most articles have evaluated short-term performance changes after exposure to new teaching or learning styles [4-7]. There is only one study that analyzed the performance differences among medical students under different systems through the objective structured clinical examination (OSCE) [8]. The first cohort of medical students in the new system graduated in 2019 and began their postgraduate year (PGY) training course. Hence, this curricular reform experience can provide medical educators with an opportunity to compare the clinical competency between the previous and new systems of medical education.

### Objectives

The purpose of this study was to examine the clinical performance differences of postgraduate residents receiving different educational systems using standardized serial OSCEs, to identify which educational system is more suitable for medical students.

## Methods

### Ethics statement

This study was approved by the Institutional Review Board of Taipei Veterans General Hospital (2022-07-031BC), and the informed consent requirement was waived.

### Study design

This was a 2 groups pre-and post-test non-synchronized study. It was described according to the STROBE (Strengthening the Reporting of Observational studies in Epidemiology) statement available from: https://www.strobe-statement.org.

### Setting

OSCEs have been implemented in medical institutions in Taiwan since 2006 [9]. Both examiners and simulated patients who participate in OSCEs are trained to obtain a certification. The examiners rate each examinee based on a checklist and provide feedback immediately after the exam. In this study, there were 3 identical stations, including delivering bad news, assessment of abdominal pain in pregnant women, and central venous catheter placement in the pre- and post-tests. The OSCEs in August 2020 served as a pre-test to assess the performance of the participants after their university medical education. The OSCEs in July 2021, which were held after about a year of PGY training, were described as the post-test. Of the 101 residents, 78 were trained in the new medical education system, and the remaining 23 were trained in the previous medical education system in college. The PGY training program was identical for all the study subjects between the pre- and post-tests. We used these 3 stations to assess examinees’ clinical reasoning, communication skills, and procedural skills, respectively. The scoring items in the “clinical reasoning” included the assessment of abdominal pain, history taking, pelvic examination, and medical management in pregnant women. The scoring items in “communication skills” were delivering bad news and explanation of illness. The “procedural skills” were scored on preparation, sterilization, manipulation, and removal of the central venous catheter. The full scores of the 3 stations were 20, 20, and 24 points, respectively. In the context of the global coronavirus disease 2019 pandemic in 2021, we used video calls on the post-test to replace the simulated patients on the pre-test to comply with the government’s social distancing policies.

### Participants

From 2020 onwards, residents in the PGY program at Taipei Veterans General Hospital, one of the largest tertiary medical centers in Taiwan, underwent an OSCE pre-test at the beginning of their training and a post-test approximately 1 year later. A total of 101 PGY residents participated in OSCEs at the beginning of their training (August 2020) and approximately 1 year later (July 2021). The scenarios used on the post-test of OSCE were the same as those used on the pre-test. Basic participant information, including gender, age, school of graduation, and graduation grades, was also collected by administrators participating in this study. This information was presented to the statistician after replacing the examinees’ names with codes to avoid the disclosure of personal information.

### Variables

The outcomes used to assess the performance of the examinees in this study were the total score and the pre- and post-test improvements. For simplicity of presentation, the full score for each station was standardized to 100 points. The improvement was calculated by subtracting the pre-test score from the post-test score of the item and dividing it by the pre-test score. The degree of progress was presented as a percentage.

### Data sources/measurement

The examiners scored the students’ performance using a computer program, and the results were automatically processed. All variables were recorded in an Excel spreadsheet (Microsoft Corp., Redmond, WA, USA).

### Bias

No bias was found in the study scheme.

### Study size

This study was not intended to determine an effect, and it was therefore not indicated to calculate the sample size.

### Statistical methods

This study analyzed the differences in OSCE performance among participants educated in different academic systems. We also analyzed whether age, gender, university attended, and school performance affected OSCE scores. As the sample size was relatively small and the number of examinees varied among groups, the Mann-Whitney U test was used to analyze score differences, and the results were expressed as median and interquartile range (IQR). Statistical analyses were performed using IBM SPSS ver. 26.0 (IBM Corp., Armonk, NY, USA). Statistical significance was set at a P-value <0.05.

## Results

### Participants

**Fig. 1.**
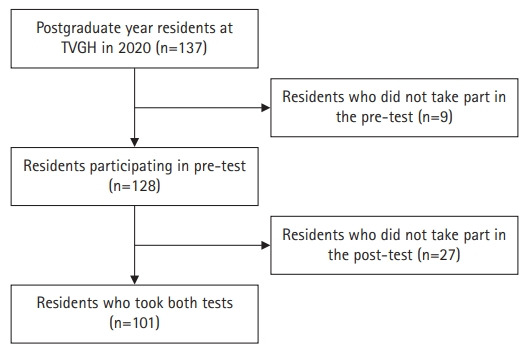
Flowchart for the inclusion of participants. TVGH, Taipei Veterans General Hospital.

In August 2020, a total of 137 residents entered the PGY training program. Nine residents did not take the pre-test for personal reasons, whereas 29 residents did not take the post-test for work and personal reasons. The number of residents who took both the pre-test and the post-test was 101 (Fig. 1).

### Main results

The baseline characteristics of the participants are shown in Table 1. Data from 101 PGY residents were available for analysis, with 78 participants educated through the new curriculum in university and the other 23 participants educated through the previous one. Participants’ ages ranged from 24 to 38 years old (median [IQR], 26 [2]); 65 (64.36%) of them were men, and 55 (54.46%) had graduated from public universities. The participants’ academic performance at university was divided into 4 groups based on quartiles. Out of 101 participants, 72 (71.28%) had grades in the top 50% of their universities. There were no significant differences in gender and university attended between the 2 groups that were educated in the new and previous medical education programs. Participants who were educated in the previous system were generally older than those who were educated in the new system (median [IQR], 27 [1] vs. 25 [1]; P<0.001). Regarding academic performance, participants educated in the new system had significantly higher scores at university (P=0.032).

Table 2 presents the pre- and post-test scores of OSCEs for the 2 groups that learned in different educational programs. In the pre-test, examinees who were educated in the new system scored higher (median [IQR], 50 [15]) in clinical reasoning than those who were educated in the previous system (median [IQR], 40 [10]; P<0.001). There was no significant difference between the 2 groups in terms of communication and procedural skills. For the total scores of the 3 stations, examinees who were educated in the new system scored significantly higher than those who were educated in the previous system (median [IQR], 199.59 [33.75] vs. 182.5 [25]; P=0.019). In the post-test, examinees who learned in the new educational program only scored higher in “history taking,” an item that assesses clinical reasoning (median [IQR], 25 [10] vs. 20 [10]; P=0.033). No differences were observed in communication skills, procedural skills, or total scores on the post-test.

**Fig. 2.**
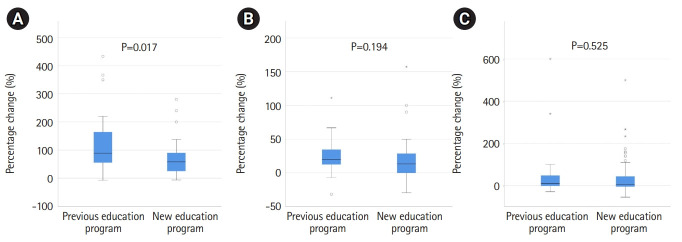
(A) Percentages of improvement in the pre- and post-test scores of clinical reasoning. The improvement percentages of the previous educational program group were significantly greater than those of the new education group (median [interquartile range], 88.89% [115.87%] vs. 57.78% [65%]; P=0.017). (B) Percentages of improvement in the pre- and post-test scores of communication skills. The previous educational program group tended to improve more than the new educational program group, but this difference did not reach statistical significance. (C) Percentages of improvement in the pre- and post-test scores of procedural skills. The previous educational program group tended to improve more than the new educational program group, but this difference did not reach statistical significance.

The post-test results of both groups in all 3 stations showed significant improvements compared to the pre-test (all P<0.05). The improvement percentages of the 3 stations are shown in Fig. 2. In clinical reasoning (Fig. 2A), the improvement percentages of the students who learned in the previous educational program were significantly greater than those of the students who learned in the new program (median [IQR], 88.89% [115.87%] vs. 57.78% [65%]; P=0.017). In terms of communication skills (Fig. 2B), procedural skills (Fig. 2C), and the total scores of the 3 stations, the students educated in the previous system tended to improve more than those educated in the new system, but these differences did not reach statistical significance.

In addition to analyzing the effects of different education programs on OSCE performance, we also assessed the effects of other factors, including age, gender, university attended, and academic performance, on OSCE performance. For age, no differences were seen in the 3 stations in either the pre- or post-test.

On the pre-test, male participants outperformed female participants in communication skills (median [IQR], 75 [15] vs. 70 [8.75]; P=0.005). Clinical reasoning and procedural skills on the pre-test showed no significant differences according to gender. Further, the 3 stations tested on the post-test revealed no significance for gender. As for the improvement rate of post-test scores in the 3 stations and the total scores, there was no significant difference between men and women.

In terms of university attended, whether students attended a public or private medical school had no effect on their OSCE scores in the pre- or post-test. In academic achievement, interestingly, participants with lower scores in university had better performance in procedural skills on the pre-test (median [IQR], 87.5 [39.58] in the bottom quarter; 79.17 [27.08] in the lower middle quarter; 79.17 [20.83] in the upper middle quarter; and 62.5 [39.58] in the top quarter; P=0.008). However, participants with higher scores in university performed better in clinical reasoning on the pre-test (median [IQR], 50 [17.5] in the top quarter vs. 40 [25] in the bottom quarter; P=0.017). On the post-test, there were no significant differences in the OSCE scores among the 4 groups with different academic performances. As for the improvement percentage, participants in the top quarter had a higher improvement rate in procedural skills (median [IQR], 28.58% [122.60%] in the top quarter; 0% [29.28%] in the upper middle quarter; 0% [31.05%] in the lower middle quarter; and 0% [50.94%] in the bottom quarter; P=0.006). There was no significant difference among the 4 groups in the improvement rates of clinical reasoning, communication skills, and the total scores of the 3 stations.

## Discussion

### Key results

In this study, we found that students educated in the new system performed better in clinical reasoning on the pre-test than students educated in the previous system. In terms of the total scores of the 3 stations of the pre-test, those who were educated in the new system also scored significantly higher. In the post-test, there was no difference in the total scores between the groups of students who were educated in the new and previous systems. After 1 year of PGY training, residents educated in the previous system improved significantly more in clinical reasoning than those educated in the new system.

### Interpretation

The new curriculum was designed to strengthen students’ active learning skills to develop medical professionals’ lifelong learning, independent thinking, and problem-solving skills [8]. The actual approaches included promoting whole-person education, reducing didactic lectures, increasing problem-based learning hours, introducing the block system, an integrated teaching method based on organ category, and providing medical students with early opportunities for primary care during their clerkship [10,11]. Although the new curriculum takes only 6 years, 1 year less than the previous curriculum, the residents’ performance on OSCEs was not worse, and may have even been better on some of the pre-tests. We believe that the new program group’s improved performance in clinical reasoning on the pre-test was reflective of the emphasis on training medical students to develop their thinking skills.

### Comparison with previous studies

Clinical reasoning is an essential skill for all physicians and can lead to the correct diagnosis, treatment planning, and patient safety. Clinical reasoning has been recognized as a subject of science since the 1950s, and many medical educators are studying distinct ways to enhance clinical reasoning for medical students or junior physicians [12]. A systematic review published in 2021 reported that 12 of the 17 articles showed performance improvements among medical students after the intervention [13]. The teaching or learning styles included in these 12 articles included structured reflection, self-explanation, generating differential diagnoses, case presentation techniques, workshops with illness scripts, and schemas. A study by Okubo et al. [7] showed that the performance of students who received team-based learning (TBL) instruction in clinical reasoning was superior to that of students who did not receive TBL instruction. Yang et al. [6] demonstrated in a pilot study that small-group tutoring courses could intensify the clinical reasoning of medical students. The subjects in this study were exposed to new teaching styles for a longer period of time than the subjects in the previous studies. It is noteworthy that the post-test in our study showed no difference in performance between residents educated in the previous system and those educated in the new system after approximately 1 year of PGY training. We believe that this illustrates the importance of continued postgraduate medical training for clinicians.

In addition to the curriculum structure, we analyzed the impact of age, gender, university attendance, and academic performance on pre- and post-test performance. On the pre-test, we found that men performed significantly better than women in communication skills. A study conducted in the 1980s showed that male doctors tended to be more active and controlled in their communications. Notably, in research, relationships between gender and communication are often inconclusive [14]. This study used the “delivering bad news” scenario to assess communication skills. This scenario was likely to be more conducive to men demonstrating their qualities of communication. After 1 year of clinical training, there was no significant difference in communication skills between men and women on the post-test. This suggests that clinical training could improve communication skills and reduce possible innate differences.

Regarding procedural skills, we found that residents with lower overall scores in medical school performed better on the pre-test, while residents with higher scores in school scored lower. The results suggest that procedural skills are less assessed in Taiwan’s medical education assessment system.

### Limitations/generalizability

This study had some limitations. First, this was not a controlled study. The participants in this study came from different universities. The differences in education methods between universities were inevitable. Second, the number of participants in this study was relatively small, which may have resulted in the relatively low power of 0.58 in this study, but there were still some significant differences between the 2 groups. As the 7-year curriculum had passed into history, there were only a few residents who had graduated from the previous program at our single center. Further multi-center studies in the future are needed to confirm these results. Third, the scenarios used on the post-test of OSCE were the same as those used on the pre-test. Although some participants may have memorized the test questions, the 2 tests were already approximately 1 year apart. The test questions were not released after the examinations and the participants did not know whether the questions would be the same or not between the pre- and post-tests. This may still offer a fair and equal standard between the 2 tests with the same scenarios.

### Suggestion

We believe our experience can serve as a guide for future studies on the effectiveness of changing academic structures in medical schools.

### Conclusions

This study demonstrates that the new medical education program has achieved its goal of improving the thinking and problem-solving skills of residents. Through postgraduate training, the clinical competencies of students undergoing the previous program were not inferior to those of the new program.

## Figures and Tables

**Table 1.** Baseline demographics between the previous and new education programs

Characteristic	All (n=101)	Previous program (n=23)	New program (n=78)	P-value
Gender, male (n, %)	65 (64.36)	18 (78.26)	47 (60.26)	0.113
Age (yr) (IQR)	26 (2)	27 (1)	25 (1)	<0.001
University, public (n, %)	55 (54.46)	12 (52.17)	43 (55.13)	0.803
Academic performance (n, %)				0.032
Top quarter	37 (36.63)	9 (39.13)	28 (35.90)	
Upper middle quarter	35 (34.65)	5 (21.74)	30 (38.46)	
Lower middle quarter	21 (20.79)	4 (17.39)	17 (21.79)	
Bottom quarter	8 (7.92)	5 (21.74)	3 (3.85)	

**Table 2.** Scores of the pre-test and post-test in postgraduate year residents from different educational programs

Tested items	Pre-test		P-value	Post-test		P-value
	Previous program (n=23)	New program (n=78)		Previous program (n=23)	New program (n=78)	
Clinical reasoning	40 (10)	50 (15)	<0.001	80 (20)	85 (15)	0.279
Communication skills	75 (15)	75 (15)	0.423	90 (10)	90 (15)	0.473
Procedural skills	70.83 (29.17)	75 (26.04)	0.916	83.33 (16.67)	83.33 (20.84)	0.829
Total	182.5 (25)	199.59 (33.75)	0.019	254.17 (33)	252.9 (43.54)	0.939

Values are presented as median (interquartile range).

## Supplementary materials

Supplement 1. Audio recording of the abstract.
